# Multi-omics prediction of key proteases regulating scar formation and neural repair after spinal cord injury

**DOI:** 10.4103/NRR.NRR-D-25-01391

**Published:** 2026-01-27

**Authors:** Huan Jian, Jiahao Ren, Jiawei Du, Kailin Wu, Shen Liu, Yuanting Zhao, Hengxing Zhou, Shiqing Feng

**Affiliations:** 1 Department of Orthopedics, Tianjin Medical University General Hospital, International Science and Technology Cooperation Base of Spinal Cord Injury, Tianjin Key Laboratory of Spine and Spinal Cord, Tianjin, China; 2 Department of Spine Surgery, Honghui Hospital, Xi’an Jiaotong University, Xi’an, Shaan Province, China; 3 Department of Orthopedics, Qilu Hospital of Shandong University, Cheeloo College of Medicine, Shandong University, Jinan, Shandong Province, China; 4 Shandong University Center for Orthopedics, Advanced Medical Research Institute, Cheeloo College of Medicine, Shandong University, Jinan, Shandong Province, China; 5 Center for Reproductive Medicine, Shandong University, Jinan, Shandong Province, China; 6 Department of Orthopedics, The Second Hospital of Shandong University, Cheeloo College of Medicine, Shandong University, Jinan, Shandong Province, China

**Keywords:** bulk RNA sequencing, fibrous scar, machine learning analysis, neuroimmunology, neuroinflammation, neurological function, protease, single-cell RNA sequencing, spinal cord injury, therapeutic drug

## Abstract

Neurological injury is often accompanied by extensive infiltration of macrophages along with activation of fibroblasts and endothelial cells. The activity of these cells is associated with elevated levels of various proteases, which contribute to the hydrolysis of multiple proteins, disrupt the extracellular matrix, and further promote the migration of immune cells into uninjured neural tissue. In this study, we combined single-cell sequencing with bulk RNA sequencing data from spinal cord injury to identify up-regulated protease-related differentially expressed genes post-injury. Using gene set variation analysis, least absolute shrinkage and selection operator regression, and random forest methods, we identified adamalysins, serine proteases, and matrix metalloproteinases as key protease types. Weighted gene co-expression network analysis combined with machine learning algorithms helped predict critical protease genes involved in spinal cord injury. Immune infiltration and single-cell analyses were applied to identify cell types enriched in proteases and their spatial localization. Molecular docking and *in vivo* and *in vitro* assays using a mouse model of spinal cord injury were used to validate potential drug interactions. We identified Mmp12 and Adam17 as key effectors regulating injury progression, and determined that macrophages, fibroblasts, and monocytes are the primary cells mediating the functions of core proteinases after injury. Subsequent *in vivo* and *in vitro* experiments demonstrated that selective inhibition of key protease activity with marimastat reduced axonal demyelination and fibrous scar formation after spinal cord injury, thereby promoting the recovery of neurological function. Our study identified the key proteases that regulate spinal cord injury repair along with their mechanisms of action, and verified that inhibiting these proteases effectively alleviates scar formation and inflammatory cell infiltration, providing novel therapeutic targets for the treatment of spinal cord injury.

## Graphical abstract

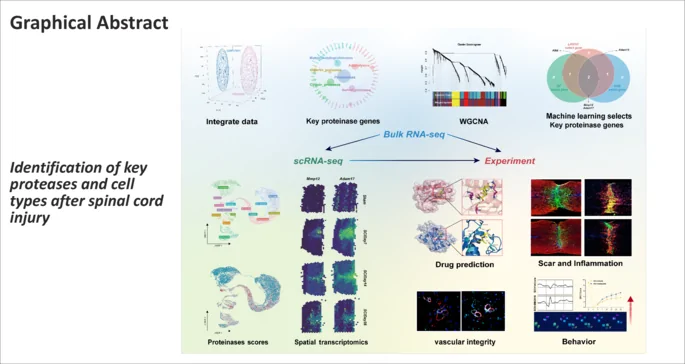

## Introduction

Traumatic spinal cord injury (SCI) predominantly results from high-impact trauma such as that occurring in vehicular accidents, falls, or violence. Traumatic SCI is accompanied by a series of complex pathological processes, including tissue ischemia, cell death, and inflammatory responses (Jain et al., 2015; Fan et al., 2022). Cell death and axonal demyelination result in the production of a large amount of cellular and myelin debris (Alizadeh et al., 2019), which stimulates bone marrow-derived macrophages to migrate, engulf, and phagocytize it, eventually transforming them into a foam-like macrophage phenotype (Van Broeckhoven et al., 2021). Compared with microglia, the infiltrating macrophages are less effective at processing the engulfed debris and lipids, which leads to persistent extracellular stimulation, further exacerbating immune infiltration and neural tissue damage (Greenhalgh and David, 2014). Various pathological processes ultimately cause changes in the spinal cord tissue structure, with the formation of scars and cystic cavities (Tator, 1995; Sofroniew, 2018). This destruction of normal tissue structure makes neural repair difficult after SCI, resulting in permanent neurological damage.

**Highlights**

• Multi-omics analysis identified Mmp12 and Adam17 as key protease genes involved in the pathological process of spinal cord injury.

• Macrophages, fibroblasts, and monocytes are the primary cell types enriched with proteases after spinal cord injury, and they are concentrated at the injury site.

• Inhibition of the activity of key proteases can effectively alleviate axonal demyelination and fibrous scar formation following spinal cord injury.

Astrocytes, microglia, and fibroblasts all participate in the formation of scar tissue following SCI (Klapka and Müller, 2006; Sofroniew, 2014; Adams and Gallo, 2018). Early after injury, astrocytes are activated under the influence of various inflammatory and chemotactic factors, forming reactive astrocytes (Sofroniew, 2014). These reactive astrocytes, along with microglia, form a protective glial barrier that isolates the injured spinal cord from uninjured tissue, thereby preventing expansion of the injury zone (Bellver-Landete et al., 2019). Although astrocytes involved in the formation of dense scars hinder axonal regeneration in the middle to late stages of SCI, the fibrotic core of the scar originates from the extracellular matrix (ECM) produced by activated fibroblasts (Soderblom et al., 2013). Under chronic inflammatory stimulation, abnormally deposited ECM forms at the injury center, resulting in fibrotic scars that are a major cause of obstructed neural growth (Fawcett et al., 2012; Harris et al., 2017).

Neural system injuries are often accompanied by significant infiltration of macrophages and activation of fibroblasts and endothelial cells. The activity of these cells is related to levels of various proteinases that can be primarily classified into four major structural families: matrix metalloproteinases (MMPs), a disintegrin and metalloproteinases (ADAMs), cysteine cathepsins, and serine proteinases (Barrett, 1992; Chapman et al., 1994; Urbich et al., 2005; Wu et al., 2018). Proteinases participate in the hydrolysis of multiple proteins, thus disrupting the ECM and further facilitating the migration of immune cells into uninjured neural tissues (Wareham and Calkins, 2025). Cells located at the injury center produce ECM and proteinases, facilitating the remodeling of neural structures. ECM remodeling is the pivotal driver of scar formation after neural injury (Lau et al., 2013); once damage occurs, the lesion site undergoes an inflammatory reaction, ECM turnover, and proliferation of glial and fibroblast populations, ultimately producing glial and fibrotic scars. The ECM not only supplies structural support but also modulates cell behavior through receptor-mediated signals (Luo et al., 2022; Sun et al., 2024). Tissue-associated proteinases reshape the ECM by degrading collagen, laminin, fibronectin, and other components, thereby influencing scarring and axon regeneration (Shechter et al., 2011; Zhu et al., 2015). Additionally, following neural injury, extracellular proteinases exacerbate blood–brain barrier damage because of their degradative effects on structural proteins like tight junction proteins and fibronectin (Gingrich and Traynelis, 2000; Chelluboina et al., 2015a; Marcos-Contreras et al., 2016). Myelin basic protein, which is important in the nerve myelination process, can serve as a substrate for various proteinases (such as serine proteinases and MMPs), and excessive accumulation of proteinases after central nervous system injury leads to the degradation of myelin basic protein, resulting in axonal demyelination (Casella et al., 2020).

Despite significant progress in understanding the molecular mechanisms of SCI, several critical gaps remain. First, while much of the current research has focused on individual proteinases involved in SCI pathology, the relative contributions of different proteinases to the post-injury inflammatory response and tissue remodeling have not been fully explored (Zhang et al., 2011), and a comprehensive analysis comparing the roles of these proteinases in SCI pathology is lacking. Second, while individual genes linked to proteinase activity have been identified, a comprehensive genome-wide screen capturing the full spectrum of genes involved in proteinase-related pathways and identifying key shared or central regulators has yet to be performed (Chao et al., 2013; Remacle et al., 2018). Third, the interactions between different proteinases and immune cells in the SCI microenvironment remain poorly understood. The complex interplay between proteinase activity, immune cell infiltration, and scar formation suggests that further investigation into these interactions could reveal new regulatory mechanisms and therapeutic targets.

Addressing these gaps calls for a more integrated approach to studying proteinases and their role in SCI pathology. Therefore, this study combines single-cell sequencing data with whole-transcriptome sequencing data of SCI to detect the differential expression of various proteinases at different time points post-injury, and explores correlations between proteinase activity, immune cell infiltration, and scar formation. By means of machine learning approaches, we predicted the key proteinase genes involved in the pathological process of SCI, and then by using molecular docking analysis technology, we screened small-molecule compounds capable of effectively regulating these key proteinase genes. Ultimately, this study attempted to achieve effective regulation of scar formation and neural regeneration after SCI by selectively intervening with the activity of these key proteinases.

## Methods

### Dataset acquisition

This study used four publicly available datasets retrieved from the Gene Expression Omnibus (GEO). Specifically, mRNA expression profiles were obtained from datasets GSE5296 and GSE47681, single-cell transcriptomic data were obtained from GSE162610, and spatial transcriptomic data were obtained from GSE195783. After extracting control samples and samples from days 1, 3, and 7 post-thoracic SCI in mice, the dataset GSE47681 included a total of 34 samples (9 control and 25 SCI) and GSE5296 included 17 samples (8 control and 9 SCI). All datasets were used in accordance with the original data usage policies.

### Data processing and gene identification for key proteinases

All statistical analyses were carried out using R software (version 4.1.3, https://www.r-project.org/). To correct for batch effects arising from the integration of the bulk RNA sequencing datasets GSE5296 and GSE47681, the ComBat function within the sva package (version 3.42.0, https://bioconductor.org/packages/release/bioc/html/sva.html) was applied. The effectiveness of batch correction was assessed and visualized through principal component analysis (PCA) using the FactoMineR (version 2.4, https://www.cran-e.com/package/FactoMineR) and factoextra (version 1.0.7, https://cran.r-project.org/web/packages/factoextra/index.html) packages in R.

Genes that were differentially expressed between the control and SCI groups were determined using the limma package (version 3.46.0, https://bioinf.wehi.edu.au/limma/) (Ritchie et al., 2015), with selection criteria set at |log2(fold change)| > 1 and *P* < 0.05. Overlaps between ferroptosis-related genes and the identified differentially expressed genes were subsequently explored and visualized using the UpSetR package (version 1.4.0, https://cran.r-project.org/web/packages/UpSetR/index.html).

Key proteinases in SCI were identified using the least absolute shrinkage and selection operator (LASSO) method through the glmnet package in R. The coefficients for the variables in the LASSO model were: Matrix_metalloproteinases, 2.098298; Adamalysins, 3.575021; Serine_proteases, 2.430833; Cystein_proteases, 0; Aspartic_proteases, 0.46292. Following this step, the random forest (RF) algorithm from the randomForest package (version 4.7-1, https://www.rdocumentation.org/packages/randomForest/versions/4.7-1) was employed to build an ensemble model based on multiple decision trees, thereby improving model accuracy and narrowing down potential proteinases. Proteinases that were identified in the LASSO model and had a mean reduction in Gini scores above 2 in the RF model were considered key proteinases.

### Weighted gene co-expression network analysis

We identified co-expressed gene modules associated with protease activity through weighted gene co-expression network analysis (WGCNA), thereby providing a research perspective complementary to differential analysis at the network level. WGCNA was performed using the WGCNA package (v1.70-3, https://rdocumentation.org/packages/WGCNA/versions/1.73; Langfelder and Horvath, 2008). This method is robust and can be used to identify gene modules and construct gene co-expression networks, facilitating the exploration of key regulatory genes.

An appropriate soft-thresholding power (set to six) was determined using the pickSoftThreshold function, and based on the topological overlap matrix (TOM), genes with similar expression patterns were classified into different modules using average linkage hierarchical clustering and dissimilarity measures. A minimum module size of 20 was set (minModuleSize = 20), and modules were merged at a cutHeight of 0.05. The associations between each module and the scores for different types of proteases were calculated, and the module with the highest correlation was prioritized for subsequent analyses to ensure its biological relevance to the target phenotype.

### Gene ontology enrichment analysis

GO enrichment analysis was performed using the clusterProfiler R package (v4.4.4, https://bioconductor.org/packages/release/bioc/html/clusterProfiler.html) with the org.Mm.eg.db database. The steps performed in this analysis were: target gene IDs were converted to Entrez ID to ensure consistency with the annotation database ID type; the enrichGO function was used with parameters OrgDb = org.Hs.eg.db, keyType = “ENTREZID”, ont = “ALL” (covering BP/CC/MF), pAdjustMethod = “fdr”, qvalueCutoff = 0.05, and pvalueCutoff = 0.05; results were filtered to retain significantly enriched GO terms with a *Q*-value < 0.05, and enrichment was visualized via dotplot (bubble size = gene count, color = adjusted *P*-value).

### Single-gene set variation analysis and immune infiltration analysis

Gene set variation analysis (GSVA) was performed using the GSVA package (version 1.42.0, https://www.rdocumentation.org/packages/Seurat/versions/4.1.0; Hänzelmann et al., 2013). For this analysis, a background gene set consisting of proteinase-related genes was used, and proteinase scores were computed for each sample through the GSVA function.

To investigate immune cell infiltration, the xCell package (version 1.1.0, https://github.com/dviraran/xCell/) was employed to estimate the relative abundances of 17 distinct spinal cord cell types across all samples. Additionally, leveraging feature genes of each cell type derived from single-cell transcriptomic data, the ssGSEA algorithm was applied to quantify the infiltration levels of these 17 spinal cord cell types within each sample. Visualization of cellular infiltration patterns in spinal cord tissues was carried out using the pheatmap package (version 1.0.12, https://cran.r-project.org/web/packages/pheatmap/index.html). Finally, associations between ridge scores derived from the ridge regression model and the estimated abundances of spinal cord cell types were evaluated using Spearman correlation analysis implemented via the ggcor package (version 0.9.8.1, https://github.com/hannet91/ggcor).

### Machine learning–based identification of key regulator genes associated with proteinases following spinal cord injury

The glmnet package (version 4.1-3, https://www.rdocumentation.org/packages/glmnet/versions/4.1-3) was used to identify critical regulator genes linked to proteinase activity after SCI and to construct a predictive model. A support vector machine-recursive feature elimination (SVM-RFE) algorithm was executed using the caret package. The LASSO algorithm was applied to select proteinase genes that were differentially expressed between SCI and control conditions (Friedman et al., 2010).

Following this, the RF approach was implemented using the randomForest package. This ensemble learning technique aggregates multiple decision trees to enhance predictive performance and can refine the selection of potential biomarker candidates (Liaw and Wiener, 2002). Genes that were identified as overlapping across the LASSO, SVM-RFE, and RF (with a mean Gini reduction > 2) models were classified as key genes.

Using these key proteinase-associated regulator genes, a ridge regression model was constructed using the glmnet function by setting the alpha parameter to zero. The ridge score for the proteinases was calculated according to the following formula: proteinases ridge score = 1.7998 × Adam17 + 0.5624 × Mmp12 – 8.6521. Model performance was assessed by plotting receiver operating characteristics (ROC) curves for the GSE47681 and GSE5296 datasets using the pROC package (version 1.18.0, https://www.rdocumentation.org/packages/pROC/versions/1.18.0).

### Single-cell RNA sequencing and spatial transcriptomics

Analyses of the single-cell transcriptomic dataset GSE162610 were conducted using the Seurat package (version 4.1.0, https://www.rdocumentation.org/packages/Seurat/versions/4.1.0). To represent cellular clusters in two dimensions, we applied uniform manifold approximation and projection (UMAP) for dimensionality reduction. Differential gene expression analyses were carried out using the FindAllMarkers function combined with the MAST statistical method.

To assess the similarity between single-cell and bulk RNA sequencing data, we utilized the SCISSOR package (version 2.0.0, https://sunduanchen.github.io/Scissor/vignettes/Scissor_Tutorial.html; Sun et al., 2022). Pearson correlation coefficients were calculated to evaluate associations between individual cells and bulk samples. A regression-based framework was employed to refine the phenotype-related correlation matrix, enabling the high-confidence selection of cells that shared transcriptional characteristics linked to specific phenotypes.

For spatial transcriptomic analysis, we first performed PCA using the RunPCA function in Seurat. Subsequently, spatial clustering and resolution enhancement of individual spatial spots were achieved via the spatialCluster function within the BayesSpace workflow. Finally, analyses of cell-to-cell communication networks were carried out and visualized using the CellChat package (version 1.6.1, https://rdrr.io/github/sqjin/CellChat/; Jin et al., 2021).

### Molecular docking analysis

To identify potential drug candidates interacting with core genes, enrichment analysis was conducted using drug–gene interaction information from the DSigDB database (https://dsigdb.tanlab.org/DSigDBv1.0/; Yoo et al., 2015) as the reference gene set. The two-dimensional chemical structures of the selected compounds were retrieved from the PubChem database (https://pubchem.ncbi.nlm.nih.gov/), while the corresponding protein structures were sourced from UniProt (https://www.uniprot.org/).

Using AutoDock4 software (version 4.2.6, https://github.com/ccsb-scripps/AutoDock-GPU), docking simulations were carried out in which proteins were treated as rigid receptors and small molecules were allowed flexibility during the docking process, with all structures prepared in PDBQT format. Visualization of the docking interactions and binding conformations was subsequently performed with PyMOL software (version 2.2.0, https://pymol.org/), and the optimal conformation with the lowest binding scores (the smaller the value, the higher the stability) was screened out.

### Animals

Adult female C57BL/6J mice (8–10 weeks old, weighing 18–20 g) were purchased from SPF (Beijing) Biotechnology Co., Ltd. (Beijing, China; license No. SCXK (Jing) 2019-0010). The mice were age- and weight-matched and housed under controlled conditions at 21–26°C with regular ventilation. All animal experiments were approved by the Animal Ethics Committee of Tianjin Medical University General Hospital (Tianjin, China; approval date: November 25, 2021; approval No. IRB2021-DW-74) and complied with the ARRIVE 2.0 guidelines (Percie du Sert et al., 2020). After acclimatization, the mice were randomly divided into three groups (sham-operated group, SCI + vehicle group, and SCI + marimastat group) using a computer-generated random number sequence, with 10 mice per group.

### Pharmacological inhibition of proteinases and establishment of the spinal cord injury model

Marimastat (Cat# M2699) was purchased from Sigma-Aldrich (St. Louis, MO, USA) as a colorless powder with a high-performance liquid chromatography purity greater than 98%. For *in vivo* experiments, mice received intraperitoneal injections of marimastat at a dose of 9 mg/kg starting 1 day before SCI and continuing daily for 14 days.

The SCI contusion model was established as previously described (Li et al., 2020). Following anesthesia with isoflurane (induction concentration: 5%; maintenance concentration: 2%; RWD Lifescience, Shenzhen, China, R510-22-10), a laminectomy was performed to fully expose the spinal cord at the T10 level, ensuring complete visualization from one side to the other. The spinal cord was then compressed for 2 seconds using 0.1-mm forceps. After the procedure, the muscle and skin were carefully sutured. Each mouse received an intraperitoneal injection of 0.5 mL of saline and was placed in a 37°C incubator until recovery from anesthesia.

### Behavioral assessment

Motor function was evaluated using the Basso Mouse Scale (Basso et al., 2006) by two independent investigators who were blinded to the treatment groups. Behavioral assessments were performed preoperatively and 1, 3, and 7 days post-injury, followed by weekly evaluations until the end of the experiment. At 28 days post-injury, motor evoked potentials were recorded with an electrophysiological device (YRKJ-G2008, Yiruiikeji, Shanghai, China). Motor evoked potential latency and amplitude were quantified to assess neurological function. Additionally, gait analysis was conducted using the CatWalk XT system (Noldus, Beijing, China). Behavioral parameters, including the regularity index and paw placement positions, were quantified using the CatWalk XT software.

### Immunofluorescence staining

The mice were anesthetized with sodium pentobarbital (60 mg/kg) and then euthanized by cervical dislocation. Spinal cord sections from injured mice were fixed with 4% paraformaldehyde and dehydrated through a sucrose gradient solution. The spinal tissue was embedded in optimal cutting temperature compound and sectioned into 10-µm slices. After blocking with 5% bovine serum albumin for 1 hour, the sections were incubated overnight at 4°C with primary antibodies, followed by five washes with Tris-buffered saline with Tween 20 (TBST), each lasting 6 minutes. The sections were then incubated with secondary antibodies at room temperature for 2 hours, followed by five additional washes with TBST, each lasting 6 minutes. Finally, the samples were observed under a fluorescence microscope (Olympus, Tokyo, Japan). For the quantitative analysis of fluorescence intensity, we specifically analyzed only the region within 1 mm from the SCI epicenter. The primary antibodies used included rabbit anti-collagen I (1:200; Abcam, Cambridge, UK, Cat# ab270993, RRID: AB_2927551), rat anti-CD68 (1:400; Abcam, Cat# ab53444, RRID: AB_869007), rabbit anti-myelin basic protein (1:400; Abcam, Cat# ab218011, RRID: AB_2895537), mouse anti-CSPG (1:200; Millipore, Billerica, MA, USA, Cat# MAB5284, RRID:AB_2219944), rat anti-CD31 (1:200; Abcam, Cat# ab7388, RRID:AB_305905), rabbit anti-NeuN (1:200; Abcam, Cat# ab177487, RRID:AB_2532109), and mouse anti-NEFH (1:200; Thermo Fisher Scientific, Waltham, MA, USA, Cat# 13-1300, RRID:AB_2532999). The secondary antibodies, all sourced from Abcam, included fluorescent Alexa Fluor 488-conjugated goat anti-mouse (1:200, Abcam, Cat# ab150113, RRID: AB_2936985), Alexa Fluor 555-conjugated goat anti-rabbit (1:200, Abcam, Cat# ab150078, RRID: AB_2544613), and Alexa Fluor 555-conjugated goat anti-rat (1:200, Abcam, Cat# ab150158, RRID: AB_3101870).

### Western blot assay

Spinal cord tissue within a 5-mm radius around the injury epicenter was harvested after injury. The tissue was lysed in radioimmunoprecipitation assay lysis buffer, followed by homogenization and centrifugation to collect the protein supernatant. Target proteins were separated on a 4%–20% gradient gel (EpiZyme, Shanghai, China, #LK309) and transferred to a polyvinylidene fluoride membrane (Millipore, #IPVH00005). The membrane was blocked with 5% non-fat milk for 1 hour and then incubated overnight at 4°C with primary antibodies. After three washes with TBST (10 minutes each), the membrane was incubated with secondary antibodies at room temperature for 1 hour, followed by three additional washes with TBST (10 minutes each). Protein bands were visualized using ECL reagent (Millipore, #WBKLS0100) and were imaged with the Tanon-5200 imaging system (Tanon, Shanghai, China). Densitometric analysis was performed using ImageJ software (National Institutes of Health, Bethesda, MD, USA; Schneider et al., 2012), and the protein expression levels were normalized to β-actin as the internal reference. The primary antibodies used included rabbit anti-β-actin (1:1000; Cell Signaling Technology, Boston, MA, USA, Cat# 4970, RRID: AB_2223172), mouse anti-Occludin (1:1000, Thermo Fisher Scientific, Cat# 33-1500, RRID: AB_2533101) and rabbit anti-Claudin-5 (1:1000; Cell Signaling Technology, Cat# 49564, RRID: AB_3065250). The secondary antibodies (all from Beyotime, Shanghai, China) included HRP-labeled goat anti-rabbit (1:1000, Cat# A0208, RRID: AB_2892644) and HRP-labeled goat anti-mouse (1:1000, Cat# A0216, RRID: AB_2860575).

### Statistical analysis

Statistical analysis was conducted using GraphPad Prism (version 9.5.0, GraphPad Software, Boston, MA, USA, www.graphpad.com). For multiple comparisons, either one-way or two-way analysis of variance with Bonferroni correction was applied, as appropriate. Basso Mouse Scale scores were evaluated with two-way repeated-measures analysis of variance followed by Bonferroni’s *post hoc* test. Differences in single variables between two groups were assessed using Student’s *t*-test. Data are presented as mean ± standard error of the mean (SEM).

## Results

### Identification of key proteinases associated with spinal cord injury

**Figure 1:**
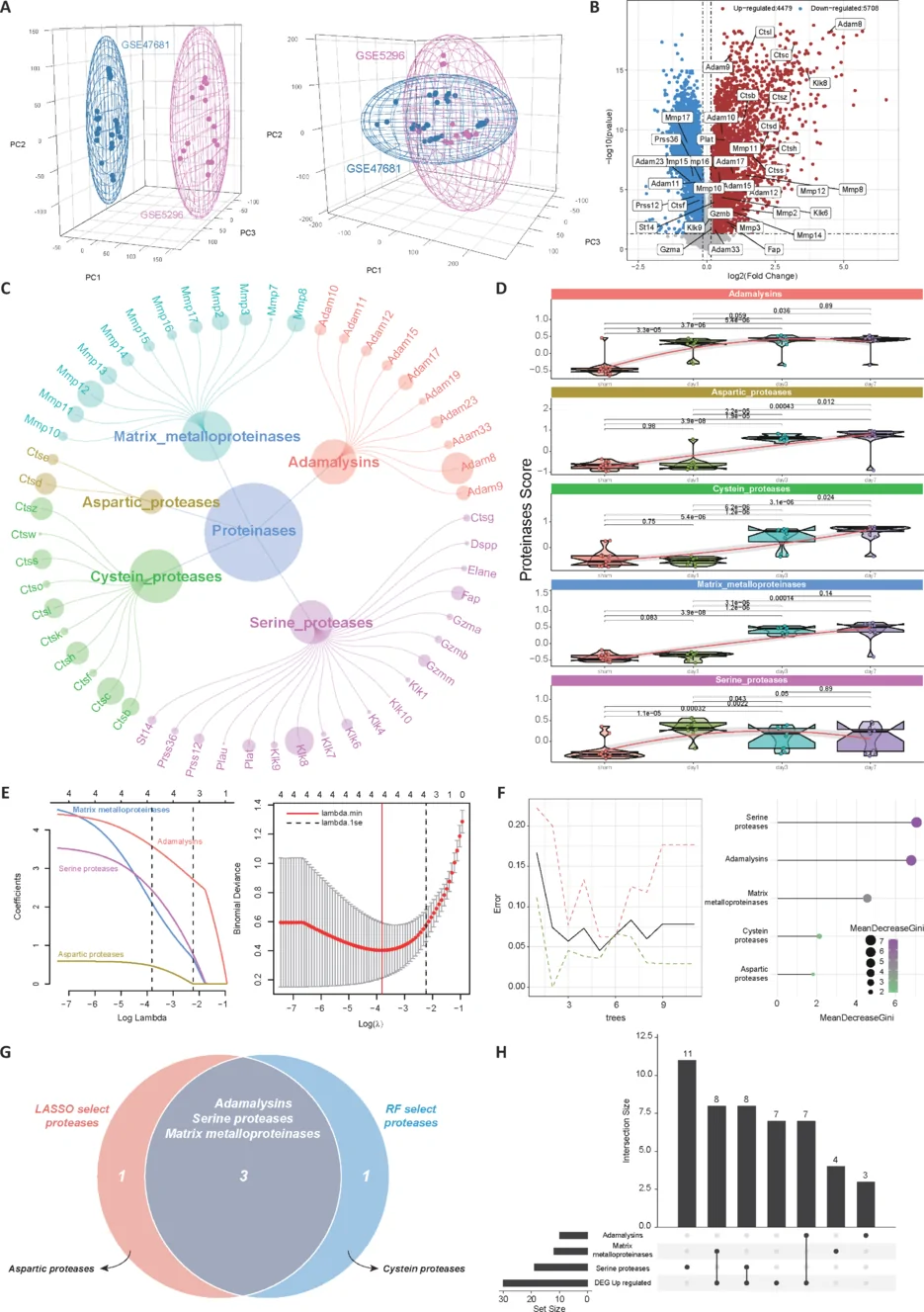
Identification and analysis of key proteinases in SCI. (A) PCA plot of the clustering of GSE47681 and GSE5296 datasets before (left) and after (right) batch effect correction using SVA. (B) Volcano plot of DEGs in SCI samples. (C) Gene interaction network of five proteinases: adamalysins, serine proteinases, matrix metalloproteinases, aspartic proteinases, and cystein proteinases. (D) Violin plots of the expression score of each proteinase calculated by GSVA, demonstrating gradual upregulation post-SCI. (E) LASSO regression analysis identifying key proteinases involved in SCI pathology. (F) Random Forest algorithm ranking the importance of proteinases in SCI. (G) Venn diagram showing the overlap of proteinases selected by LASSO and random forest algorithms. (H) Upset plot of the intersection of upregulated DEGs and selected proteinases. DEGs: Differentially expressed genes; GSVA: gene set variation analysis; LASSO: least absolute shrinkage and selection operator; PCA: principal component analysis; SCI: spinal cord injury.

To expand the sample size and achieve robust results, we integrated data from different sources. We downloaded the GSE47681 and GSE5296 datasets and used the sva package to correct for batch effects. PCA confirmed the effectiveness of batch correction (**Figure 1A**). Next, differential expression analysis was conducted to identify genes that are differentially expressed during SCI, with a focus on the proteinase genes we collected (**Figure 1B**). We specifically examined proteinase genes that were upregulated during SCI and highlighted five proteinase classes with tissue-dissolving functions, namely MMPs, adamalysins, aspartic proteases, cysteine proteases, and serine proteases (**Figure 1C**).

GSVA software was used to calculate the scores for each proteinase class, which revealed that the expression of these proteinases gradually increased after SCI (**Figure 1D**). We then performed LASSO regression and RF methods to identify key proteinases involved in SCI (**Figure 1E** and **F**). This revealed that adamalysins, serine proteinases, and MMPs play crucial roles after SCI (**Figure 1G**). Additionally, we found multiple gene intersections between upregulated differentially expressed genes and these three proteinase classes (**Figure 1H**).

### Gene co-expression and key module identification of key proteinases associated with spinal cord injury

**Figure 2:**
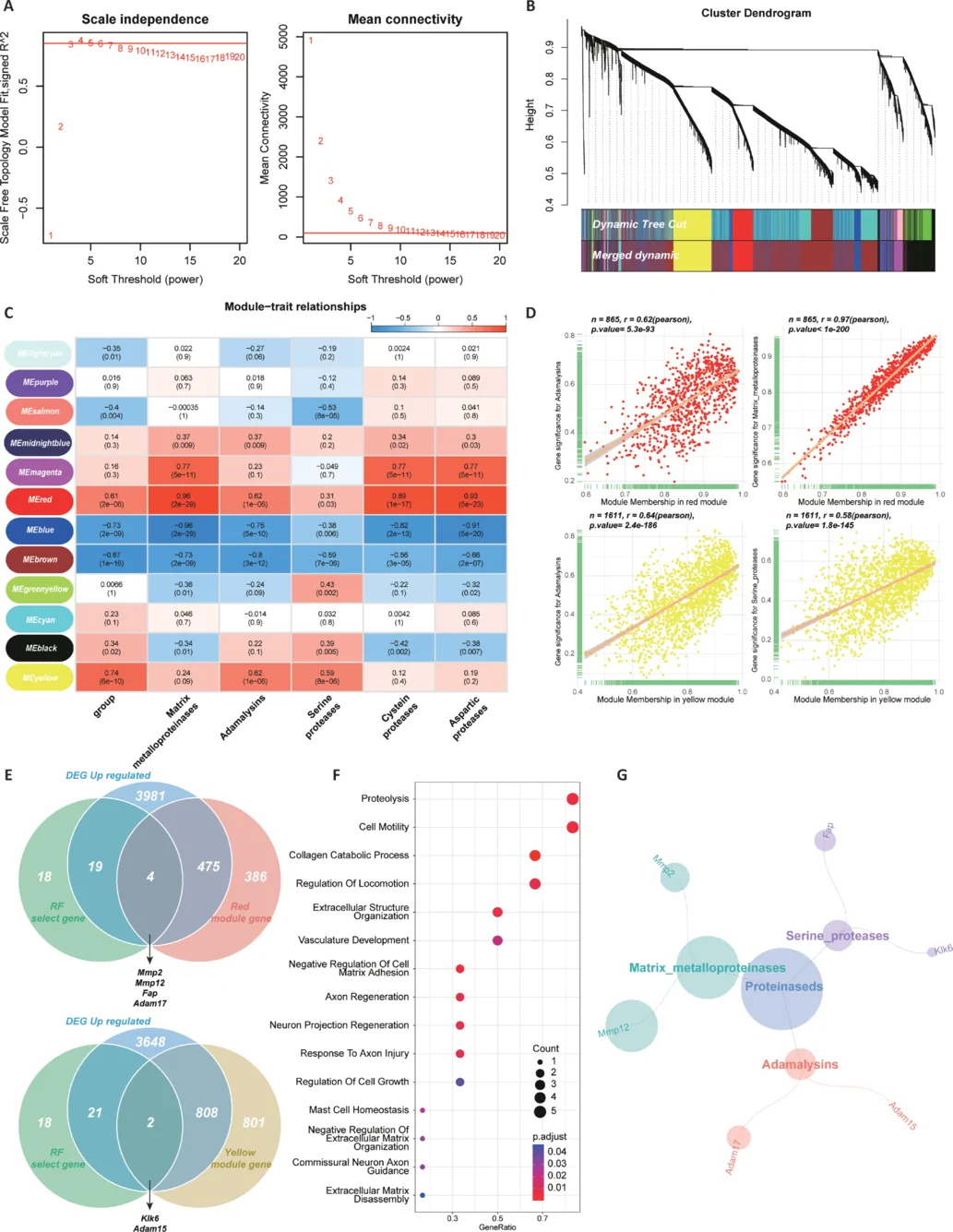
WGCNA module identification and functional analysis of key proteinases. (A) Scale-free topology fit index and mean connectivity plots of WGCNA. (B) Hierarchical clustering of WGCNA modules. (C) Module-trait correlation heatmap of the relationships between modules and proteinases. (D) Scatter plots of the module membership in the red and yellow modules, which were associated with adamalysins and matrix metalloproteinases (red module) and adamalysins and serine proteinases (yellow module). (E) Venn diagram of the intersection of upregulated DEGs and selected genes from the red and yellow modules. (F) GO enrichment analysis of the intersected genes, highlighting pathways related to matrix remodeling, scar formation, and neural regeneration. (G) Gene network visualization of the relationships between the six selected genes and their associated proteinases. DEGs: Differentially expressed genes; GO: Gene Ontology; WGCNA: weighted gene co-expression network analysis.

On the basis of the scale-free network structure, a soft threshold of three was chosen for the network construction, as it was the lowest power for which the scale-free topology fit index (*R*^2^) reached 0.85 (**Figure 2A**). Hierarchical clustering analysis of gene co-expression was performed, resulting in 16 modules. Using dynamic tree cutting with a height of 0.1, we identified 12 modules that each had a unique gene expression pattern (**Figure 2B**). The relationship between the modules and proteinases was further examined (**Figure 2C**), revealing the modules that most strongly correlated with proteinases. In the module-feature correlation heatmap shown in **Figure 2C**, in which the 12 different modules are given different colors, the red and yellow modules are associated with multiple proteinases. Specifically, the red module is linked to adamalysins and MMPs, while the yellow module is associated with adamalysins and serine proteinases (**Figure 2D**). Based on these findings, we selected the red and yellow modules for further analysis.

We then intersected these modules with the three key proteinase classes (adamalysins, serine proteinases, and MMPs) and upregulated differentially expressed genes, identifying Mmp2, Mmp12, Fap, and Adam17 from the red module, and Klk6 and Adam15 from the yellow module (**Figure 2E**). GO enrichment analysis revealed that these genes were significantly enriched in pathways related to matrix remodeling, scar formation, and regeneration, suggesting their roles in tissue remodeling and neural regeneration following SCI (**Figure 2F**). Finally, we constructed a network to visualize the relationships of these six genes with the three proteinase classes (**Figure 2G**).

### Identification of core proteinase genes associated with spinal cord injury and immune infiltration analysis

**Figure 3:**
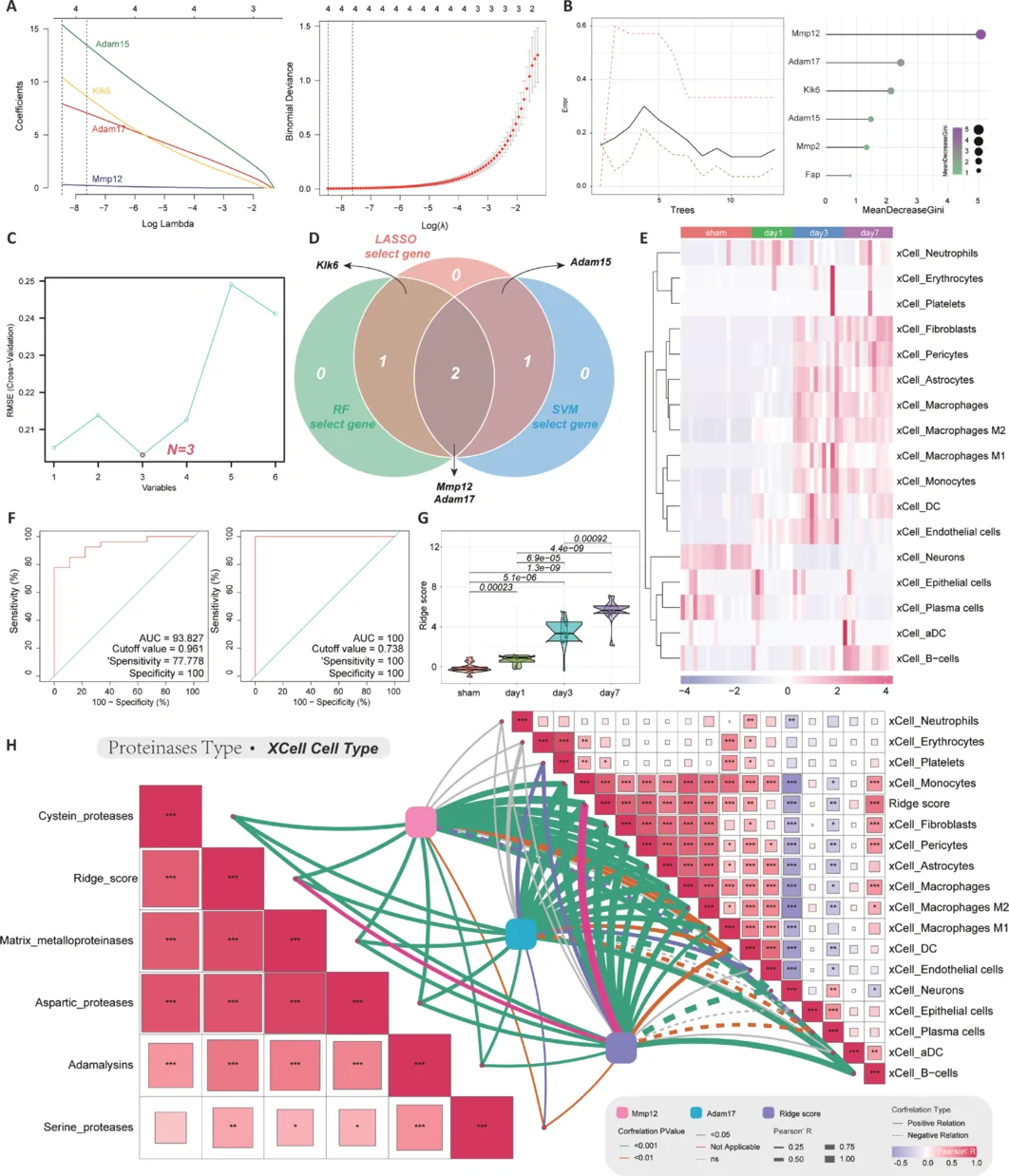
Machine learning-based identification of key proteinase genes and immune infiltration. (A–C) LASSO regression, random forest, and SVM-RFE analyses for identifying key proteinase genes. (D) Venn diagram of gene selection across algorithms. (E) xCell-based immune infiltration analysis showing temporal changes in different types of immune cells after SCI. (F) Ridge regression model for Mmp12 and Adam17, validated in both training and validation datasets. (G) Ridge score dynamics over time post-SCI. (H) Correlations heatmap between ridge regression score, proteinases, and immune cells. *P < 0.05, **P < 0.01, ***P < 0.001. Adam17: A disintegrin and metalloproteinase 17; AUC: area under the curve; LASSO: least absolute shrinkage and selection operator; Mmp12: matrix metalloproteinase 12; SCI: spinal cord injury; SVM-RFE: support vector machine-recursive feature elimination.

For all samples, 70% were randomly selected as the training set and the remaining 30% were used as the validation set. Key genes were identified using three machine learning algorithms: LASSO regression, RF, and SVM-RFE (**Figure 3A–C**). After integrating the results from the three algorithms, Mmp12 and Adam17 were determined to be the key proteinase genes involved in SCI (**Figure 3D**).

Cell type enrichment-based immune infiltration analysis of the SCI transcriptomic data performed using xCell revealed that fibroblasts, macrophages, monocytes, and pericytes showed varying levels of enrichment over time following SCI (**Figure 3E**). A ridge regression score model was constructed based on the key genes Mmp12 and Adam17, and the reliability of this regression model was validated in both the training and validation datasets (**Figure 3F**). The regression score showed a gradual increase over time post-SCI (**Figure 3G**) and the model showed strong associations with all proteinases and significant associations with the infiltration of monocytes, fibroblasts, macrophages, astrocytes, and other cell types (**Figure 3H**).

### Identification of proteinase-related cell types in single-cell analysis

**Figure 4:**
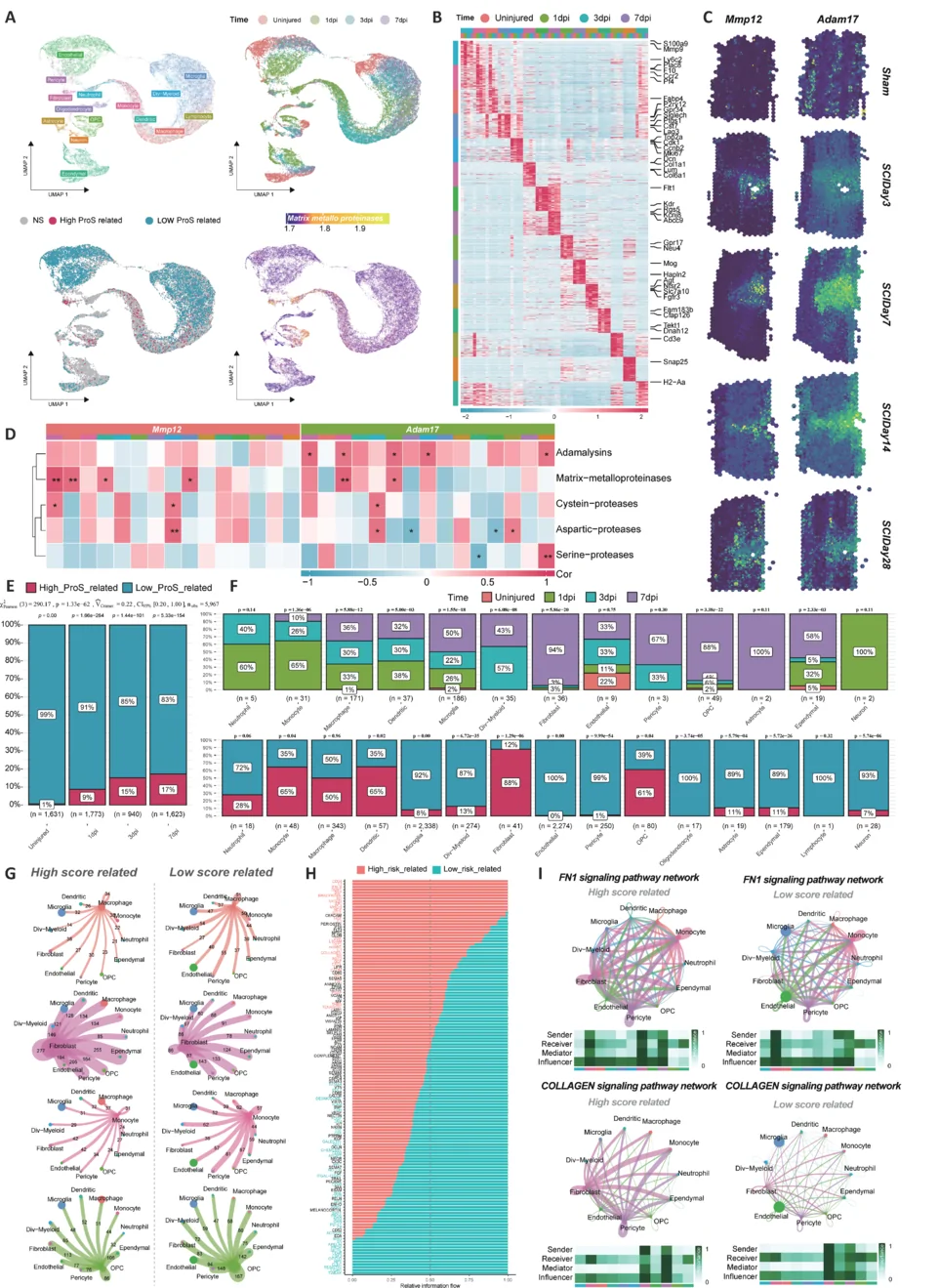
Identification of proteinase-related cell types and their role in scar formation. (A) UMAP plot of single-cell RNA sequencing data in cell types associated with high and low proteinase scores. (B) The heatmap displays the specific gene information of different cell types at various time points post-SCI. (C) Spatial transcriptomic analysis of the expression patterns of Mmp12 and Adam17 at the SCI injury site, with higher expression levels at the injury center persisting for up to 28 days. (D) Correlation matrix of the relationship between proteinase scores and the expression of Mmp12 and Adam17 in different cell types. *P < 0.05, **P < 0.01. (E) The distribution of cells in the high and low ProS groups at different time points post-SCI. (F) Proportions of different immune cells at various time points post-SCI (uninjured, 1, 3, and 7dpi) (top) or in the high and low ProS groups (bottom). (G) Cell–cell communication network analysis of the intensity of communication in fibroblasts, macrophages, monocytes, OPCs and other cells in the high/low ProS group. (H) Relative information flow of genes associated with high and low FeS-related pathways. (I) Comparison of Fn1 and collagen signaling pathways between high and low ProS groups. Adam17: A disintegrin and metalloproteinase 17; dpi: day(s) post-injury; Fn1: fibronectin 1; Mmp12: matrix metalloproteinase 12; OPC: oligodendrocyte precursor cell; ProS: proteinase score; SCI: spinal cord injury; UMAP: uniform manifold approximation and projection.

To further identify the cell types showing significant differences in proteinase expression following SCI, we analyzed the single-cell RNA sequencing data from GSE162610. Using cell-specific gene markers, spinal cord cells were classified into different subpopulations (**Figure 4A** and **B**). First, we divided all SCI samples into high proteinase score (ProS) and low ProS groups using the ridge regression score based on bulk data. Then, using SCISSOR software, we identified the cell types associated with these two groups. We found that cells associated with the high ProS group were mainly fibroblasts, monocytes, dendritic cells, and macrophages (**Figure 4A**, bottom).

To examine the spatial distribution of Mmp12 and Adam17 expression after SCI, we analyzed spatial transcriptomic data. We found that regions with significantly increased expression of Mmp12 and Adam17 were located in the center of the SCI site, and that this expression persisted for up to 28 days post-injury. This suggests that these regions, where matrix remodeling and scar formation occur, are closely associated with the injury center (**Figure 4C**).

By calculating the correlation between proteinase scores and the expression of Mmp12 and Adam17 in different cell types, we found that Mmp12 and Adam17 play major roles in regulating post-SCI proteinase levels in various cell types, especially fibroblasts, macrophages, and monocytes. This indicates that targeting the expression of specific genes in these cell types could potentially inhibit the activity of these proteinases (**Figure 4D**). Quantification of the cells associated with the high and low ProS groups identified by the SCISSOR software revealed that the increase in ridge regression scores after SCI primarily concerned fibroblasts, monocytes, dendritic cells, and macrophages (**Figure 4E** and **F**). Immune communication analysis showed that the number and strength of communications were higher in fibroblasts related to the high ProS group (**Figure 4G**). Further comparison of the communication differences between the two groups revealed that fibronectin 1 (Fn1) and collagen signaling were significantly higher in cells from the high ProS group than in those from the low ProS group (**Figure 4H** and **I**), confirming the involvement of Mmp12 and Adam17 in scar formation after SCI.

### Prediction and validation of proteinase-specific inhibitors

**Figure 5:**
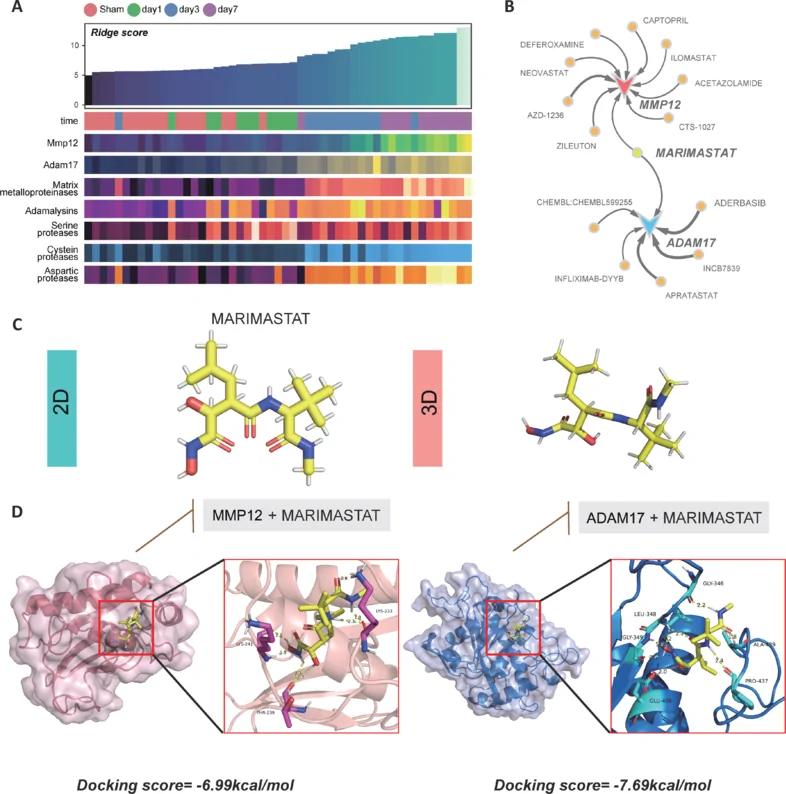
Identification and validation of inhibitors targeting proteinases. (A) Ridge regression scores for Adam17 and Mmp12 across all samples. (B) Network visualization of small molecule inhibitors identified through DSigDB. (C) 2D and 3D molecular structures of marimastat. (D) Molecular docking results showing binding interactions of marimastat with Mmp12 and Adam17. 2D: Two-dimensional; 3D: three-dimensional; Adam17: a disintegrin and metalloproteinase 17; DSigDB: drug signatures database; Mmp12: matrix metalloproteinase 12.

The proteinase ridge regression scores for Adam17 and Mmp12 were ranked across all samples, and high consistency was confirmed between the five proteinase types and regression scores (**Figure 5A**). Using the DSigDB database, marimastat was identified as a small molecule inhibitor that effectively interacts with both Adam17 and Mmp12 (**Figure 5B**). The two- and three-dimensional structures of marimastat are shown in **Figure 5C**. Molecular docking experiments further verified that marimastat can effectively bind to Mmp12 and Adam17, with binding scores lower than −5 kcal/mol (**Figure 5D**). To assess the biological effects of the predicted proteinase inhibitor and the impact of proteinase regulation on SCI repair, we next established an SCI mouse model and evaluated tissue repair and motor function post-injury.

### Proteinase inhibition impedes scar formation and promotes motor function recovery after spinal cord injury

**Figure 6:**
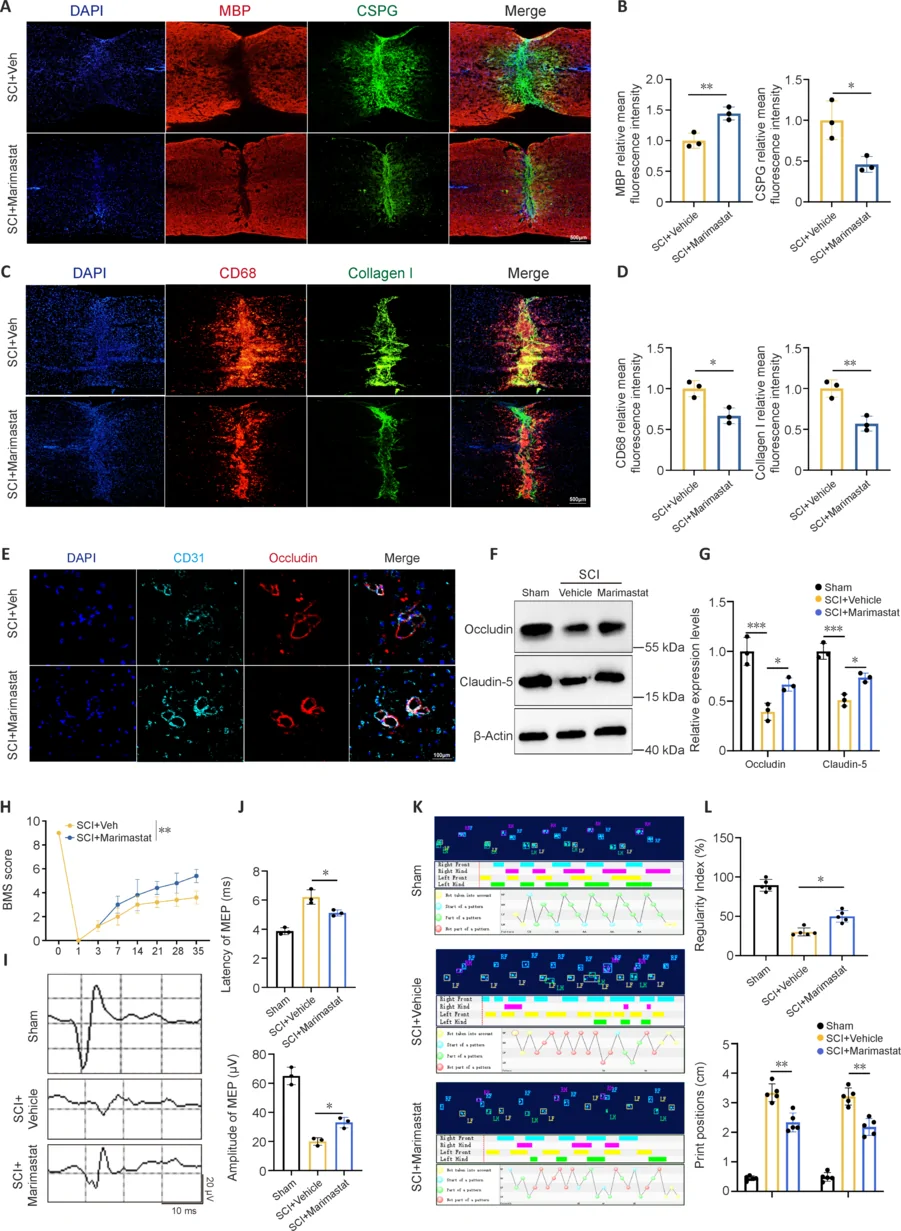
Inhibition of key proteases effectively reduces scar formation and promotes functional recovery after SCI. (A) Immunofluorescence images of the expression of CSPG (green, Alexa Fluor 488) and MBP (red, Alexa Fluor 555) at the injury site 14 days post-SCI. Compared with the SCI + vehicle group, the SCI + marimastat group exhibited increased MBP fluorescence intensity at the site of injury, along with decreased CSPG fluorescence intensity. Scale bar: 500 μm. (B) Quantification of CSPG and MBP fluorescence intensity (*n* = 3). (C) Immunofluorescence staining of CD68^+^ (red, Alexa Fluor 555) cell and collagen I (green, Alexa Fluor 488) expression at the injury site. Compared with the SCI + vehicle group, the SCI + marimastat group showed a significant reduction in the fluorescence intensity of both CD68 and Collagen I at the site of injury. Scale bar: 500 μm. (D) Quantification of CD68^+^ myeloid cell infiltration and collagen I expression (*n* = 3). (E) Immunofluorescence staining of occludin (red, Alexa Fluor 555) and CD31 (cyan, Alexa Fluor 488) expression at the injury site. Compared with the SCI + vehicle group, the SCI + marimastat group showed a significant increase in the fluorescence intensity of both CD31 and occludin at the injury site. Scale bar: 100 μm. (F) Western blot analysis of occludin and claudin-5 levels in the injured spinal cord. (G) Quantification of occludin and claudin-5 protein levels (*n* = 3). (H) BMS score of the SCI + vehicle and SCI + marimastat groups within 35 days post-injury (*n* = 5). (I) Representative MEPs. (J) Quantification of MEP latency and amplitude (*n* = 3). (K) CatWalk gait analysis. (L) Quantification of the regularity index and paw placement positions of CatWalk results (*n* = 5). Data are presented as mean ± SEM. *P < 0.05, **P < 0.01, ***P < 0.001 (two-sided Student’s *t*-test [B, D], one-way analysis of variance with the Bonferroni *post hoc* test [G, J, L], or two-way repeated measures analysis of variance with Bonferroni *post hoc* test [H]). BMS: Basso Mouse Scale; collagen I: type I collagen; CSPG: chondroitin sulfate proteoglycan; DAPI: 4’,6-diamidino-2-phenylindole; MBP: myelin basic protein; MEP: motor evoked potential; SCI: spinal cord injury.

Immunofluorescence analysis of mouse spinal cord at 14 days post-injury revealed significantly lower fluorescence intensities of CSPG and Collagen I in the marimastat-treated group compared with the vehicle group (**Figure 6A–D**), indicating that proteinase inhibition effectively hindered scar formation after SCI. Additionally, marimastat alleviated damage to axons and myelin structure caused by excessive proteinases (**Figure 6A**, **B**, and **Additional Figure 1**). The reduction in ECM destruction significantly decreased the immune infiltration of CD68^+^ myeloid cells into the injury center (**Figure 6C** and **D**). Marimastat effectively prevented the degradation of tight junction proteins and the disruption of blood vessel structures at the injury borders that would otherwise be caused by MMP12 and ADAM17 (**Figure 6E**). Western blot analysis further confirmed the rescue of occludin and Claudin-5 levels in injured spinal cord tissues, with marimastat preventing the significant decrease in tight junction protein levels (**Figure 6F** and **G**). Both vehicle- and marimastat-treated mice exhibited normal motor behavior prior to SCI. At 1 day post-injury, both groups were almost completely paralyzed. However, although the vehicle group gradually regained motor function after SCI, the marimastat-treated mice showed better recovery than the vehicle group (**Figure 6H**). Post-injury, mice displayed prolonged latency and significantly reduced motor evoked potential amplitudes. Marimastat treatment effectively improved bioelectrical signals following SCI (**Figure 6I** and **J**) and enhanced hindlimb coordination, as reflected by the regularity index and footprint position (**Figure 6K** and **L**). These findings suggest that inhibition of MMP12 and ADAM17 proteinases promotes functional recovery after SCI.

## Discussion

Scar formation severely restricts axonal regeneration and functional recovery after SCI, and suppressing this process is therefore a major therapeutic goal. Immediately after SCI, a local inflammatory response clears cellular debris and confines the lesion, but excessive inflammation drives scar development (Zhao et al., 2022; Xu et al., 2025). The scar has two main components: a glial scar formed by reactive astrocytes and other glial cells, and a fibrotic scar generated by fibroblasts, perivascular cells, and other stromal cells. Both create physical and chemical barriers that block axon regrowth and impede neural repair (Dias et al., 2018). Experimental strategies to limit scarring include dampening inflammation, reducing astrocyte proliferation, and transplanting stem cells (Anderson et al., 2016; Hellenbrand et al., 2021; Hu et al., 2023). Nevertheless, early scar formation affords neuroprotection by containing inflammation and maintaining tissue architecture (Anderson et al., 2016). Effective therapies should therefore preserve structural integrity and immune balance while minimizing the scar’s inhibitory effects on axon regeneration.

This study identifies adamalysins, serine proteinases, and MMPs as the key proteinases involved in scar formation, demonstrating their spatiotemporal expression patterns and impacts on post-injury scar formation. WGCNA followed by cross-validation with random-forest and LASSO models singled out Mmp12 and Adam17 as key effectors of lesion progression. MMP12 is expressed primarily by macrophages and microglia. Beyond its classic elastase activity, it degrades basement-membrane components such as type IV collagen, fibronectin, and laminin, thereby facilitating macrophage infiltration during the inflammatory phase (Wells et al., 2003; Chen, 2004; Suh et al., 2012; Chelluboina et al., 2015b). Its substrate spectrum also includes N-cadherin, myelin basic protein, and pro-TNF-α, underscoring its pleiotropic functions (Veeravalli, 2024; Dorjay Tamang et al., 2025). ADAM17, a membrane-anchored disintegrin-metalloproteinase, is constitutively expressed throughout the central nervous system (Ho et al., 2022). Under normal physiological conditions it supports neural development, synaptic plasticity, and memory formation (Peschon et al., 1998). Following SCI, ADAM17 becomes highly concentrated in the lesion core, mainly within immune cells. This up-regulation promotes the shedding of pro-inflammatory cytokines such as TNF-α, leading to neuronal loss (Hsia et al., 2019). Inhibiting the iRhom2/ADAM17/TNF-α axis curbs excessive TNF-α release and mitigates macrophage-driven inflammatory damage (Haxaire et al., 2018).

Immune system infiltration into the central nervous system is a hallmark of SCI. While phagocytes help clear cellular debris and promote repair, excessive inflammation releases reactive oxygen species and cytokines that further damage neurons and glia (Hellenbrand et al., 2021; Cavalcanti et al., 2025). In the sub-acute and chronic phases of SCI, persistent inflammation enlarges the fibrotic core and suppresses axonal regrowth. Breakdown of the blood–spinal cord barrier is the main gateway for this influx of immune cells, and tissue proteinases exacerbate the breach by degrading vascular basement‐membrane components (Gao et al., 2023; Zhou et al., 2023). Our results link the extent of immune infiltration to the expression level of proteinases. Macrophages, fibroblasts, and monocytes dominated the post-injury landscape, and their abundance correlated with a ridge-regression score built on the core proteinases Mmp12 and Adam17. Both enzymes are most highly expressed in the lesion center, underscoring their functional relevance.

Screening of the DSigDB database and molecular docking pinpointed marimastat, an orally-active broad-spectrum MMP inhibitor, as a dual antagonist of MMP12 and ADAM17 (Park et al., 2020). *In vivo*, marimastat curtailed scar formation and dampened leukocyte entry into the lesion. Two mechanisms are likely to contribute to this: (i) inhibition of MMP12 and ADAM17 limits chemotactic signaling, reducing macrophage recruitment (Hsia et al., 2019); and (ii) by restraining MMP12-mediated degradation of ECM and myelin, marimastat preserves tissue architecture and diminishes secondary infiltration (Suh et al., 2012). Ischemia-reperfusion disrupts tight junction proteins such as ZO-1, claudin-5, and occludin in cerebral microvessels (Jiao et al., 2011), increasing blood-spinal cord barrier permeability and amplifying secondary damage. MMP2 and MMP9 are established mediators of claudin-5 and occludin cleavage, and MMP12 activates both enzymes (Feng et al., 2011; Kurzepa et al., 2014). Therefore, inhibition of MMP12 can help restore tight-junction integrity (Chelluboina et al., 2015b). Consistent with this paradigm, we observed that marimastat prevented the loss of claudin-5 and occludin and helped maintain microvascular integrity at the injury border.

Despite these findings, this study has several limitations. Residual batch effects may still have been present in the bulk RNA sequencing, despite SVA correction, potentially affecting the robustness of our results. AutoDock simulates single receptor–ligand pairs, and therefore marimastat could still act through additional targets and influence outcomes. Marimastat inhibits proteinases by mimicking the structure of their peptide substrates, and consequently, it does not directly alter the expression levels of ADAM17 or MMP12. We quantitatively assessed the degradation of tight junction proteins, which serves as indirect evidence of marimastat’s inhibitory effect on protease activity, but we did not directly measure its specific impact on ADAM17 or MMP12 activity. Furthermore, whether marimastat selectively targets ADAM17 and/or MMP12 within specific cell types remains a subject for future investigation. Although our findings demonstrate its ability to modulate immune infiltration and scarring, the precise molecular circuitry remains to be clarified. Finally, although the current findings of this study are statistically significant, the sample size of the sequencing data available for statistical analysis was relatively small. Future research should incorporate larger-scale raw data to improve statistical power and enhance the credibility of our conclusions.

In summary, this study combined multiple datasets and analytic approaches to chart the spatiotemporal landscape of tissue-related proteinases in SCI, and to connect the expression of these proteinases with immune infiltration and scar formation. Weighted co-expression analysis, reinforced by machine-learning cross-validation, pinpointed Mmp12 and Adam17 as core proteinase genes. Molecular docking then identified marimastat as a dual inhibitor, and *in vivo* delivery of this agent reduced scar deposition, dampened inflammatory cell entry, and preserved tight-junction proteins such as claudin-5 and occludin, thereby safeguarding microvascular integrity at the lesion border. These findings provide new insights into post-SCI structural remodeling and immune regulation, and highlight promising therapeutic targets for interventions.

## Additional file:

***Additional Figure 1:*** *Marimastat alleviates damage to both axons and myelin structure at 14 days post-SCI.*

Additional Figure 1 Marimastat alleviates damage to both axons and myelin structure at 14 days post-SCI.
Analysis of tissue samples at 14 days post-SCI demonstrated that the fluorescence intensity of NeuN (red, indicates spinal cord) and NF-200 (green, indicates myelin) in the SCI + marimastat group was significantly stronger than that in the SCI + vehicle group, and the differences were statistically significant. Scale bar: 500 μm. Data are presented as mean ± SEM (n = 3). ^*^P < 0.05, ^**^P < 0.01 (two-sided Student’s t-test). DAPI: 4',6-diamidino-2-phenylindole; NF200: neurofilament-20; SCI: spinal cord injury.

## Data Availability

*All data analyzed during this study were sourced from Gene Expression Omnibus (GEO), with accession Nos. GSE5296, GSE47681, GSE162610, and GSE195783.*
